# Supplementary material for: A fault-intrusion-tolerant system and deadline-aware algorithm for scheduling scientific workflow in the cloud
Source: PeerJ Comput Sci. 2021 Nov 2;7:e747. doi: 10.7717/peerj-cs.747 (PMC8576554; doi:10.7717/peerj-cs.747)
Supplement: Supplemental Information 6 [file peerj-cs-07-747-s006.zip › Myproject1.1/docs/org/workflowsim/clustering/class-use/BasicClustering.html]

Uses of Class org.workflowsim.clustering.BasicClustering


JavaScript is disabled on your browser.


- Overview
- Package
- Class
- Use
- Tree
- Deprecated
- Index
- Help

- Prev
- Next

- Frames
- No Frames

- All Classes

## Uses of Class org.workflowsim.clustering.BasicClustering

- Packages that use BasicClustering

  | Package | Description |
  | --- | --- |
  |  |  |
  | --- | --- |
  | org.workflowsim |  |
  | org.workflowsim.clustering |  |
  | org.workflowsim.clustering.balancing |  |
- - ### Uses of BasicClustering in org.workflowsim

    Fields in org.workflowsim declared as BasicClustering

    | Modifier and Type | Field and Description |
    | --- | --- |
    |  |  |
    | --- | --- |
    | `protected BasicClustering` | ClusteringEngine.`engine` The clustering engine to use |
  - ### Uses of BasicClustering in org.workflowsim.clustering

    Subclasses of BasicClustering in org.workflowsim.clustering

    | Modifier and Type | Class and Description |
    | --- | --- |
    |  |  |
    | --- | --- |
    | `class` | `BlockClustering` BlockClustering groups tasks in both horizontal and vertical direction |
    | `class` | `HorizontalClustering` HorizontalClustering merges task at the same horizontal level |
    | `class` | `VerticalClustering` VerticalClustering merges tasks at the same pipeline |
  - ### Uses of BasicClustering in org.workflowsim.clustering.balancing

    Subclasses of BasicClustering in org.workflowsim.clustering.balancing

    | Modifier and Type | Class and Description |
    | --- | --- |
    |  |  |
    | --- | --- |
    | `class` | `BalancedClustering` BalancedClustering is a clustering method that aims balancing task runtime and data dependency. |

- Overview
- Package
- Class
- Use
- Tree
- Deprecated
- Index
- Help

- Prev
- Next

- Frames
- No Frames

- All Classes
